# Cardiovascular Toxicity Related to Cancer Treatment

**DOI:** 10.3390/jcdd10060259

**Published:** 2023-06-14

**Authors:** Andrew Xanthopoulos, Alexandros Briasoulis

**Affiliations:** 1 Department of Cardiology, University Hospital of Larissa, 41110 Larissa, Greece; andrewvxanth@gmail.com; 2 Department of Therapeutics, Faculty of Medicine, National and Kapodistrian University of Athens, 11528 Athens, Greece

Cancer is among the major causes of death globally, accounting for nearly 10 million deaths in 2020 [1]. Advances in cancer treatment, including the development of targeted therapies, were associated with better cancer outcomes. However, the resulting increase in the number of long-term survivors was accompanied by a number of potential acute and chronic side effects that may impair the quality of life and, in some instances, the life expectancy of cancer survivors [2].

Cardio-oncology is a rapidly evolving field in which cancer patients with cardiovascular risk or disease are exposed to complex medication regimens (i.e., anthracyclines, human epidermal growth factor receptor 2 inhibitor, immune checkpoint inhibitor, tyrosine kinase inhibitor, chimeric antigen receptor T cells, etc.), placing individuals at heightened risk for potential drug interactions or adverse events such as cardiomyopathy, myocarditis, hypertension, prothrombotic events, arrhythmias, and bleeding [3,4,5]. Therefore, there is an unmet need for development and implementation of cardio-oncology programs, in order to surveil and manage cardiovascular complications before, during, and years after treatment in cancer patients. The smooth operation of such programs requires the close collaboration of health care professionals from different specialties such as oncology, cardiology, and hematology [6,7]. In this regard, Čiburienė et al. reported the 6-year experience of the first cardio-oncology service in Vilnius, Lithuania [8]. Among the 447 patients who were consulted, more than two thirds were female, and the median age was 64 years. Interestingly, early biochemical cardiotoxicity was found in 27%, early functional cardiotoxicity was observed in 17%, and early mixed cardiotoxicity was seen in 45% of referred patients treated with cardiotoxic cancer therapies.

Di Lisi et al., utilizing data from a single Italian cardio-oncology center, reported an increased rate in cancer-therapy-related cardiac dysfunction events during the COVID-19 pandemic (June–August 2021) compared to the pre-COVID-19 period (June–August 2019) [9]. The authors speculated that this finding resulted from the poor cardiological follow-up during chemotherapy at the time of the COVID-19 pandemic, as well as the reluctance of patients to visit the cardio-oncology clinic due to the risk of infection.

Wang et al. observed an association between serum inflammatory factors and cardiovascular health status in a cross-sectional study of 119 patients with non-small cell lung cancer [10]. In particular, tyrosine kinase inhibitor (TKI)-targeted drug treatment was associated with lower levels of serum leukemia inhibitory factor (LIF), reflecting underlying cardiovascular damage. Furthermore, serum TGF-1 and cardiac troponin T were found to be correlated with pre-clinical cardiovascular injury in the examined population of patients.

Dantas et al. investigated the influence of doxycycline (an anti-inflammatory and matrix metalloproteinase inhibitor) on the attenuation of doxorubicin induced cardiotoxicity, in 80 male Wistar rats (group A: control, group B: doxorubicin, group C: doxycycline and group D: doxorubicin + doxycycline) [11]. The researchers observed an increase in the activity of enzymes associated with glucose metabolism and a decrease in the activity of enzymes related to lipid metabolism (which characterizes cardiac injury) in the doxorubicin group. However, these metabolic alterations were attenuated in rats receiving doxycycline. Similarly, the study by Nancy S. Younis demonstrated the cardioprotective effects of β-caryophyllene, a natural sesquiterpene found in the essential oils of several plants, on cardiac injury induced by cyclophosphamide exposure, in 30 male Wistar rats (group 1: normal, group 2: β-caryophyllene, group 3: cyclophosphamide, group 4: β-caryophyllene + cyclophosphamide (100 mg/kg), group 5: β-caryophyllene + cyclophosphamide (200 mg/kg)) [12]. β-caryophyllene administration was associated with activated Nrf2/HO1/NQO1 and inhibited TLR4/NFkB pathways with ensuing antioxidative action and diminished inflammatory and apoptosis responses.

Georgiadis et al. reviewed the current limitations in the definition and classification of cardiotoxicity caused by chemicals, since cardiotoxicity is not limited to anticancer agents, and suggested a roadmap based on scientific evidence from animal studies, which would reduce uncertainties and bias [13]. This roadmap included: (a) identification of the appropriate animal species and strain, (b) identification of the lines of scientific evidence (e.g., histopathological, biochemical, echocardiographic indices, etc.) from animal studies with relevance to humans, (c) meta-analysis of data, (d) validation of the above-described evidence in animals exposed to other alleged cardiotoxic substances, (e) establishment of mechanisms of action, and (f) introduction of novel indices and in silico methods.

In conclusion, cardiotoxicity is an adverse reaction in chemotherapy, immunotherapy, or radiotherapy, as well as other chemical substances, and may result in poor quality of life, increased morbidity, and mortality. Implementing a cardio-oncology program to prevent, monitor, and manage cardiotoxicity in patients requiring anti-cancer treatments is both challenging and imperative (Figure 1).

## Figures and Tables

**Figure 1:**
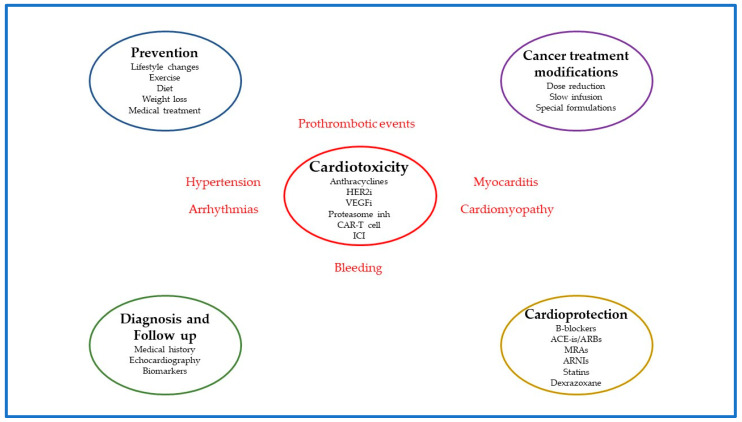
The four pillars of cardiotoxicity management in patients on anti-cancer treatments. Several anti-cancer treatments (anthracyclines, HER2i, VEGF, etc.) were associated with unfavorable cardiovascular events such as myocarditis, cardiomyopathy, hypertension, arrhythmias, prothrombotic events, and bleeding. Therefore, implementation of cardio-oncology programs to prevent, monitor, and manage cardiotoxicity in patients requiring anti-cancer treatments is crucial, and it is based on four pillars. The first pillar is prevention (both primary and secondary). Lifestyle changes such as Mediterranean diet, weight loss, and aerobic exercise along with medical treatment (antihypertensive, antidiabetic, antilipidemic) may reduce the risk of cardiotoxicity. The second pillar is diagnosis and follow up. In this regard, medical history (age, smoking, obesity, cardiovascular risk factors, heart failure, type and dose of chemotherapy), echocardiography (left ventricular ejection fraction, global longitudinal strain), and biomarkers (cardiac troponin and natriuretic peptides) should be an integral part of patient’s evaluation at the first visit and during follow-up. The third pillar encompasses the cancer treatment modifications such as the dose reduction, the slow infusion, and the utilization of special formulations of anti-cancer drugs. The fourth pillar is cardioprotection with the use of neurohormonal inhibitors (b-blockers, ACE-is, ARBs, MRAs, ARNIs), statins, and dexrazoxane. Abbreviations: HER2i, human epidermal growth factor receptor 2 inhibitor; VEGFi, vascular endothelial growth factor inhibitor; Proteasome inh, proteasome inhibitors; CAR-T cell, chimeric antigen receptor T-cell therapy; ICI, immune checkpoint inhibitors.
